# From qualitative to quantitative tractography: a novel method to measure variation and error in diffusion mr tractography datasets of the myocardium

**DOI:** 10.1186/1532-429X-13-S1-P19

**Published:** 2011-02-02

**Authors:** Choukri Mekkaoui, Shuning Huang, Guangping Dai, Timothy G Reese, Aravinda Thiagalingham, Udo Hoffmann, Marcel P Jackowski, David E Sosnovik

**Affiliations:** 1 Harvard Medical School, Charlestown, MA, USA; 2 University of São Paulo, Institute of Mathematics and Statistics, São Paulo, Brazil

## Purpose

To define a robust quantitative parameter to measure physiological variation and experimental error in diffusion MRI tractography datasets of the myocardium

## Introduction

Techniques to analyze diffusion tensor MRI (DT-MRI) datasets in the myocardium are limited. In a recent major advance, diffusion MRI tractography was used to visualize myofiber bundles as continuous 3-dimensional tracts (*Circ Cardiovasc Imaging*. 2009; 2(3):206-12). However, the tractographic scheme used in this work, while of major value, was purely qualitative. A strong need exists to develop quantitative tools for the visualization and analysis of diffusion MRI tractography datasets in the heart.

## Methods

**Figure 1:**
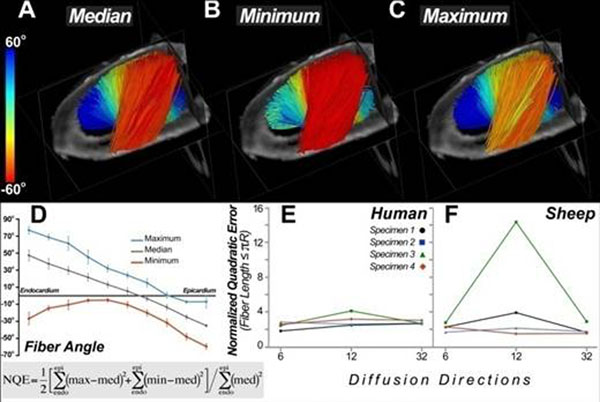
(A) Lateral view of a human heart depicting fiber tractography results from a volume of interest placed in the lateral wall. Fibers were colored according to their median helix angles. (B) Fibers within the same region colored according to their minimum helix angle values. (C) Fibers colored according to their maximum helix angles. (D) Plot of maximum, median and minimum helix values as they vary from endocardium to epicardium. (E) Normalized quadratic error (NQE) calculated for 4 human hearts imaged with 6, 12 and 32 diffusion-encoding directions. The NQE robustly detects the noisy dataset obtained in the third sheep heart (green) and shows the image quality is lowest with the 12-direction encoding scheme.

Excised human, sheep and rat hearts (n=12) were studied. Myocardial infarction was produced in the sheep hearts 3 months prior to euthanasia. DT-MRI of the human and sheep hearts was performed on a 3.0T scanner using 6, 12, or 32 gradient-encoding directions; a b-value of 2000s/mm^2^; voxel-size=2x2x2mm^3^; TR/TE=8430/96ms; and a constant acquisition duration of 30 minutes. Fiber tracking was performed with a fourth-order Runge-Kutta approach. The helix angle assigned to each continuous tract was defined by the maximum, minimum or median helix angle of the tract. The normalized quadratic error (NQE) was then defined by the quadratic error between these helix angle profiles summed across the myocardium (Figure 1D).

## Results

**Figure 2:**
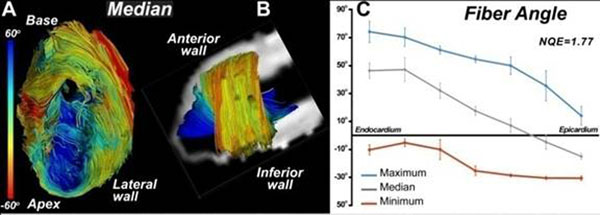
An infracted sheep heart viewed from (A) its anteriorinfarcted wall and (B) the remote zone in its lateral wall. Fibers were colored according to median helix angle values computed along individual trajectories. (C) Plot of maximum, median and minimum helix angles in the remote zone of the infarct as they vary from endocarduim to epicardium. A shift to more positive helix angle values in the remote zone as a result of the remodeling process can be observed (B,C). The normalized quadratic error (NQE) of these fibers is 1.77, consistent with a high quality dataset.

The lateral wall of a human heart is shown in Figure 1. The fiber tracts (A-C) have been color-coded according to their median, minimum and maximum values respectively. NQE values were < 5 when tract length was limited to 50% of the ventricular circumference (πR). Noisy and unstable datasets (sheep 3, panel F) were robustly detected and NQE analysis also revealed that data quality was frequently lowest with the 12-direction encoding scheme. The anterior wall of an infarcted sheep heart is shown in Figure 2A. Rightward (positive) rotation of myofiber helix angle was consistently seen in the remote zones of the infarcted sheep hearts (Figure 2B-C).

## Conclusion

A new metric (NQE) for quantifying the quality of tractography datasets in the myocardium is introduced. The technique was able to robustly differentiate high quality and noisy datasets in all 3 species. We also show tractographically, for the first time and with high confidence (low NQE), that myofibers in the remote zone of an infarct undergo a rightward rotation in helix angle. The NQE can be calculated from any tractographic dataset and is thus a highly powerful, generalizeable and translatable metric.

